# Financing the Millennium Development Goals for health and beyond: sustaining the 'Big Push'

**DOI:** 10.1186/1744-8603-6-17

**Published:** 2010-10-08

**Authors:** Gorik Ooms, David Stuckler, Sanjay Basu, Martin McKee

**Affiliations:** 1 Institute of Tropical Medicine, Department of Public Health, Nationalestraat 155, 2000 Antwerp, Belgium; 2 Oxford University, Department of Sociology, Christ Church, Meadow Flat, Oxford OXL1DP, UK; 3 London School of Hygiene & Tropical Medicine, Department of Public Health and Policy, 15-17 Tavistock Place, London WC1H 9SH, UK; 4 University of California San Francisco, Department of Medicine, and San Francisco General Hospital, Division of General Internal Medicine, 505 Parnassus Avenue, Room M987, San Francisco CA 94122, USA; 5 London School of Hygiene and Tropical Medicine, European Centre on Health of Societies in Transition, 15-17 Tavistock Place, London WC1H 9SH, UK

## Abstract

Many of the Millennium Development Goals are not being achieved in the world's poorest countries, yet only five years remain until the target date. The financing of these Goals is not merely insufficient; current evidence indicates that the temporary nature of the financing, as well as challenges to coordinating its delivery and directing it to the most needy recipients, hinder achievement of the Goals in countries that may benefit most. Traditional approaches to providing development assistance for health have not been able to address both prevalent and emergent public health challenges captured in the Goals; these challenges demand sustained forms of financial redistribution through a coordinated mechanism. A global social health protection fund is proposed to address recurring failures in the modern aid distribution mechanism. Such a Fund could use established and effective strategies for aid delivery to mitigate many financial problems currently undermining the Millennium Development Goals initiative.

## The Millennium Development Goals and the 'Charity' Paradigm

Since the Millennium Development Goals (MDGs) were established in 2000, an enormous effort has mobilized resources to reduce poverty, discrimination and disease in poor countries [1-3]. The Millennium Project's 2005 Report expected that the Goals would be achieved by the 2015 target date if high-income countries increased official development assistance from 0.25% of donor Gross Domestic Product (GDP) in 2003 to 0.44% in 2006 and 0.54% in 2015, delivering approximately US$120 billion each year of aid [4]. By 2007, the latest year with available data, that financial goal was largely met: development assistance totaled more than $120 billion, of which $22 billion was allocated to health [3].

Despite this tremendous influx of resources, many resource-poor countries continue to be unable to reach the Goals, contrary to previous projections [1,2]. About 1 billion people are likely to remain in extreme poverty in 2015, missing the poverty MDG target of halving the number of people who live on less than $1 a day by 2015. More than 1 out of every 10 school-aged children in the world remains out of school, such that the target of achieving universal primary education is unlikely to be met. Five years after the target date for eliminating gender disparity in primary and secondary education, marked disparities persist. Estimated child mortality is twice the target rate, and maternal mortality is four-times the target rate. Only some of the targets related to HIV and tuberculosis seem likely to be met [5]. Furthermore, the MDGs cover only the small share of the total burden of disease that is regularly measured in many countries, ignoring the large burden of adult disability and premature death that is increasingly driven by non-communicable diseases.

It is commonly thought that the major reason why countries cannot achieve improved health or social well-being is that they are simply too poor: poverty traps communities into a vicious cycle of inadequate capital to build schools and businesses, and such communities never have enough money to fund sustainable services needed for health, education, or other basic community infrastructure, which in turn is needed to have healthy workers and business development that would produce capital [2,6,7].

Leaders of the UN Millennium Project argued that what was needed to address this problem was an influx of capital to overcome the poverty trap (labeled by some commentators as a 'Big Push') [8]. As one leader described the strategy, "Once extreme poverty is reduced with the support of external assistance, low-income countries can begin to achieve self-sustaining economic growth on their own."[7] Thus the thinking of decision-makers in global health and development has been permeated by a heavy emphasis on a short-term increase in financing; in short, their view is that with a brief but large injection of capital, we can "help people help themselves". These are exemplary expressions of the intended temporality of global health aid:

• Ann Veneman, then executive director of UNICEF, on interventions to address child mortality: *"These plans and budgets should emphasise the continuum of care from maternal to neonatal to child survival. But they should also ensure that interventions are prioritised and phased in according to the ability of both the health system to deliver them at scale, and of governments to afford them and to sustain them in the longer term."*[9] [underline added]

• The World Health Report 2008 of the World Health Organization: *"In a significant number of these low-expenditure, low-growth health economies, particularly in sub-Saharan Africa and fragile states, the steep increase in external funds directed towards health through bilateral channels or through the new generation of global financing instruments has boosted the vitality of the health sector. These external funds need to be progressively re-channelled in ways that help build institutional capacity towards a longer-term goal of self-sustaining, universal coverage."*[10] [underline added]

• The World Bank's 'Health Financing Revisited: A Practitioner's Guide': *"Although the practical definition of fiscal sustainability may change for programs supported by the [International Monetary Fund] and [International Development Association], it is extremely unlikely that such definitions will be divorced from a country's capacity to accommodate expenditures financed with aid within the domestic budget constraint in a reasonable period of time, while maintaining sustainable levels of debt to GDP and debt service to exports."*[11] [underline added]

The 'Big Push' approach is in line with a model in which the welfare state exists at the national level but there is free trade at the international level. From this perspective, international assistance was seen as a temporary necessity: "Despite misgiving about free trade and the perceived necessity of aid to hasten economic development, many policy-makers acted on the assumption that the adoption of liberal economic policies would lead to convergence in economic performance."[12]

National welfare assistance is based on principals of communal duties and rights-the recognition that misfortunes occur perennially (natural disasters, slum development, ghettoization, trade inequalities and other perpetual creators of new or sustained poverty and bad health), and therefore so should redistribution of funds so that whole sectors of a population do not die or suffer excessively from bad fortune, as long as certain criteria can qualify individuals as rights-holders. In contrast, international assistance is temporary by definition, relying on the goodwill of countries identifying themselves as donors, even though much of the bad fortune that generates or sustains contemporary poverty is based on policies that cross borders [13]. As such, international assistance is fundamentally similar to the charity that preceded the welfare state; while within-country governance principles recognize the need for perennial redistribution, cross-border governance mechanisms do not.

## New Understandings of Slow and Unequal Progress

New evidence suggests that the global charity model, to help people help themselves temporarily rather than to create a sustained pool of redistributed funds, has provided an insufficient response to the challenges preventing MDG progress.

### The 'poverty trap' is being perpetuated rather than broken

First, the 'poverty trap' is being perpetuated rather than broken, as can be seen from the growing body of literature on this topic,[14] including work by those seeking to understand long-term growth failure in very poor countries, from the perspective of convergence and divergence in the global economy,[15,16] and from those with an interest in self-reinforcing inefficient equilibria, often linked to institutional failure, that prevent countries escaping from poverty [17]. An additional factor is lack of resilience in the face of emerging economic and environmental crises [18,19]. The UN Millennium Update report (2009) reveals that progress towards the MDGs is threatened by the recurrent reality of natural and man-made disasters creating turmoil for the poorest. Recurrent instability in the global economic marketplace has undermined the benefits of global charity repeatedly,[20] as seen previously during the Russian and Asian economic crises [21-24].

The persistence of this poverty trap has many reasons, but one fundamental etiology is the failure to support those who lose out in the creative and destructive processes of the global market [25]. In a global competitive market, strong players are allowed or even encouraged to invest their market gains in ways that give them future comparative advantage, such as education, research and development, and infrastructure: all factors contributing to further economic growth. If left unchecked, however, comparative advantages accumulate within the hands of a few families and companies, leading to further exclusion of those who were already losing out in the creative and destructive processes of the market [26]. Importantly, David Ricardo's theory of comparative advantage, the basis of modern free trade theory and the idea of trade as a mutually beneficial activity, assumed a fully competitive marketplace without the ongoing within-firm trading systems and oligopoly/monopoly or trade rule-bending mechanisms that are currently exhibited in the modern economic and political system [27]. The current system does not correct the so-called 'backwash effects' of global centers of economic growth (such as the United States, Western Europe, and increasingly Brazil, Russia, India, China, and South Africa): these centers attract capital and human resources from their remote peripheries and bend the rules to their advantage, but the fruits of their growth do not return to the periphery [26].

Within rich countries, systems have been established to protect those who have failed in the market place, for example by providing unemployment assistance to tide over those people who try to start a business but fail, enabling them to survive until they return to employment. These systems encourage or even force the most advantaged groups to share their gains with everyone else, especially those with initial disadvantages, at least to a certain extent. Through taxation and government spending, the advantages of the strongest players contribute to building up comparative advantages for the entire population, in particular by protecting those individuals who have failed in the market place. They also protect people against so-called catastrophic health expenditures, and provide early childhood education to their children, attempting to safeguard equality of opportunity. Government-encouraged measures cause so-called 'spread effects' of economic growth centers [26]. However, in a global economy, the winners and the losers are often in different countries and the system of social protection extends only to the national border: although backwash effects of economic growth centers cross borders, spread effects rarely cross borders. For every dollar of aid to Africa, about 2.4 dollars leaves Africa in the form of illicit financial flows [28]. According to other estimates, up to 40% of the private wealth of inhabitants of Africa is invested in other parts of the world--legally or illegally [29]. This capital flight is in addition to debt repayments of over $3 trillion generated when international banks lent to governments that were often directed by colonial and post-colonial dictators who were put in place to provide natural resources and labor to western states [30].

As another example of the globalised nature of risks, the recent food crisis left many poor farmers unable to sell their crops after a drop in prices (which, some economists argue, rose from the speculative actions of investors in rich countries);[20,31] these farmers lacked an insurance system that would give them time to develop a "comparative advantage" in another form of productive labor. Their countries are not only too poor to finance such initiatives; even after a large increases in aid, recurrent market failures and natural disasters can overwhelm an entire country's capital accumulation (as seen recently in Greece), requiring aid from other countries.

Current international support mechanisms, such as the International Monetary Fund, have so far failed to protect populations from the social consequences of economic instability and backwash effects, often because they are pushing countries towards becoming "self-sufficient" so quickly that they force them to reduce their remaining social welfare funds [32-35]. Short-term disadvantage accumulates--leading to chronic poverty, lack of education, lack of health care, and poor nutrition. No short-term increase in aid can counterbalance the powerful global forces that continue to create losers; aid as currently designed is therefore inevitably slated to fail.

### International assistance does not reach the poorest

Second, a recent series of reports has revealed that much aid is not reaching the poor, not just because of corrupt governments, but because of diversion of aid for other purposes and macroeconomic conditions imposed on poor country governments [34,36,37]. Based on the assumption that flows of aid are unreliable, major global financial institutions have directed Ministries of Finance to build up reserves, maintaining overall budget levels unchanged (rather than increasing the health budget when new aid is received) [38,39]. The consequences are apparent in the finding that each additional $1 dollar of health aid adds only about $0.37 to health budgets in recipient countries, and < $0.01 (complete displacement) in countries that under the advisorship of the International Monetary Fund (when analyzed using the Organization of Economic Cooperation and Development's aid database) [36,37,40].

### International assistance is often short-term

Third, aid has generally focused on brief initiatives directed at specific diseases and easily-demonstrated outcome measures that can be achieved within short time-frames, increasingly to fulfill the requirements of private donors.

### International assistance is misaligned with the actual needs of populations

Fourth, aid being allocated by donors is misaligned with the actual needs of populations, in spite of repeated donor commitments to align aid with domestic needs (a frustration captured in their decision to amend practices set out in the 2005 Paris Declaration and 2008 Accra Agenda) [41,42]. The current system of resource allocation has failed to respond to the interconnected risks that threaten progress. One example is that chronic (long-term) health problems and their underlying social and economic determinants of health are least likely to be addressed by current aid programs, yet have recently been shown to be critical determinants of progress towards the MDGs [5,43]. Furthermore, aid is volatile, and systems to pool funds to address the long-term social and economic factors most affecting MDG progress, such as the Sector-Wide Approach, have had limited success due in part to volatility and unpredictable commitments [44]. Many current donations are subject to such extensive conditionalities and earmarking as to be of limited utility, and focus so much on short-term measureable outcomes that longer-term results seem elusive.

Looking at the reality of one of the poorest countries in the world, Ethiopia, helps understanding how these challenges come together and explain difficulties achieving the MDGs:

• Ethiopia is a country of 80 million people with an average Gross Domestic Product (GDP) of US$244 per person per year and a total health expenditure level of $9 per person per year. To meet minimum health requirements related to the MDGs, the country would need to achieve a level of health expenditure of $40 per person per year according to the WHO.

• The first obstacle in meeting this need is international community support, which would have to amount to $2.4 billion per year ($30, multiplied by 80 million people), gradually decreasing in line with falling need. At present, no avenue exists to provide funds on this scale for health system development, and no aid channel has proven sustainable at such a high level given shifting interest from external donors, who already account for 42% of total health expenditure in the country.

• The second challenge for the Government of Ethiopia is to be convinced that it can sustain this level of health expenditure, from domestic revenue, within a reasonable time. That would require economic growth of more than 400% over the next 10 years, to obtain an average GDP of $1,333 per person per year in 2020; it would also require government revenue to increase to 20% of GDP ($266 per person per year) and require the allocation of government revenue to health to increase to 15% ($40 per person per year). At present, such budgetary change is at odds with international financing institution recommendations, which specify to the Ministry of Health not to increase health budgets upon receipt of new aid, given the unreliability of prior aid disbursements historically.

• This specifies the third challenge: that the major institutions making recommendations (often binding by virtue of other loan agreements) to the Ministry, particular the World Bank and the International Monetary Fund, would have to be convinced that the first and second condition will be fulfilled, otherwise the increased expenditure would not be allowed due to fears that it would lead to new building projects and initiatives that would then go bankrupt as unreliable aid dried-up (the phrase "not be allowed" comes from the World Bank's document 'Health Financing Revisited: A Practitioner's Guide') [11]. Hence, current aid systems lack of mechanism for sustainable financing to produce effective system-wide improvements to public health, even though they aim to achieve the system-wide Goals specified in the MDGs, and ultimately achieve sustainable growth of poor countries.

In light of these limitations, some critics of the current aid system suggest that the 'Big Push' approach will not solve the problems the MDGs seek to eliminate; dispirited by current obstacles, they argue aid is not part of the solution, but is becoming the problem because of its distortions of domestic priorities and interference with markets [8,45-47]. These critics suggest that progress could be achieved much faster by getting rid of aid altogether, although others have suggested that these arguments rely on distortion of data to suit pro-privatization agendas [30].

## A proposal for perennial redistribution

How can we overcome the limitations of the present development paradigm? It is prudent to investigate what has worked and to seek a pragmatic way forward.

Some of the greatest successes in global health have come from building permanent capacity through long-term channels for providing aid. The programs that are on track for MDG achievement and have proved robust in the face of continued short-term shocks are those that have coordinated and sustained funding mechanisms. Most obviously, the Global Fund to fight AIDS, Tuberculosis and Malaria (The Global Fund) has challenged the conventional notion of sustainability. When public health experts argued that only HIV prevention, not AIDS treatment, was a sustainable goal, people living with AIDS and their advocates argued that this position was simply untenable in the context of a globalized market economy that produced the migration, slum creation and gender inequalities that underpin the AIDS epidemic: they argued that reliable national *and *international funding for treatment was the only ethical approach to addressing the survival of large number of persons already infected with HIV.

As the Global Fund's executive director, Michel Kazatchkine, said in 2008: "The Global Fund has helped to change the development paradigm by introducing a new concept of sustainability. One that is not based solely on achieving domestic self-reliance but on sustained international support as well."[48] The Global Fund thus created a mechanism of redistribution of wealth between countries that is intended to be perennial. It is notable that while other MDGs remain far behind their targets, progress towards the targets for AIDS, tuberculosis and malaria has so far been more promising [1,2]. This suggests that, to create a more effective health aid system, a continuing redistribution of wealth will be needed. We are aware of the fact that the global AIDS response - and in its slipstream the global response to tuberculosis and malaria - has been supported by an exceptional mobilization of civil society groups around the world, which may not exist for wider health issues. However, we believe that the impact of this response should be convincing enough to adopt similar approaches to wider health issues and ultimately to wider social justice challenges.

### A global social health protection fund?

A proposal has been made for such a permanent system of resource redistribution through a global social health protection fund, similar to - but broader in scope than - the Global Fund [49,50]. The proposed mechanism for such a fund would be: (i) to assess contributions from member states based on a weighted burden sharing formula, (ii) provide recurrent financing to address persistent threats to health and human rights of deprived populations, and (iii) allocate resources using needs assessments so as to harmonize aid with the burden of avoidable and unfair mortality. It is not the objective of this paper to provide a detailed blueprint for a global social health protection fund, as disagreement on its features would stand in the way of broad discussion of its underlying vision. In brief, the proposal is that "some of the allocation mechanisms used by the Global Fund might be built upon by a democratized and strengthened global social governance system within the context of the sustainable resources available from a global levy based on global taxation with funds flowing through normal government budgets" [51]. A global social health protection fund could include the three abovementioned functions:

• *To assess contributions from member states based on a weighted burden sharing formula*. Ideally, the contributions would come from internationally agreed taxation, including all countries. The tax on airline tickets directed to UNITAID--a global scheme to which several developing countries are contributing--could serve as an example to be further developed [52]. In the absence of such a system, the agreed burden sharing between the donors to the International Development Association of the World Bank could be used [53]. The target would need to be in line with recent estimates about international assistance to achieve the health related MDGs, such as the ones provided by the Taskforce on Innovative International Financing for Health Systems [54].

• *To provide recurrent financing to address persistent threats to health and human rights of deprived populations*. Like any national social health protection mechanism, a global social health protection fund would have to be based on agreed rights and duties. The essential right of member states would be a right to the international assistance needed to realize the right. That presumes an agreement on the health-related goods and services that are essential for the realization of the right to health (and adapted to local realities), and an agreement on minimum domestic contribution. The Ghana Macroeconomics and Health Initiative--proposing a health systems approach based on the most prevalent causes of mortality and morbidity--can be used as a reference for essential health goods and services [55]. The Abuja Declaration can be used as a reference for the minimum domestic contribution [56]. In countries where the government is unwilling to live up to its domestic duties (as internationally agreed), civil society would be encouraged to submit its own proposals directly to the global social health protection fund, in ways similar to the present practice of civil society proposals to the Global Fund to fight AIDS, Tuberculosis and Malaria [57].

• *To allocate resources using needs assessments so as to harmonize aid with the burden of avoidable and unfair mortality*. A country-owned long-term health plan or compact would form the basis of allocations from the global social health protection fund, and for the accountability for the allocations received. On this point, the national strategies elaborated for the International Health Partnership, and the related 'joint assessment of national strategies' processes can be used as a reference,[58] if it includes civil society as the Global Fund's decisional platforms do [57]. Both processes involve many stakeholders, which would allow the global social health protection fund to remain a financing tool.

There are, of course, a number of practical issues to be addressed. One is the relationship of such a fund to existing initiatives, such as the Global Fund. Specifically, should there be a single fund or multiple ones? We believe that the interlinkages among diseases and their determinants argue for a single fund but we also recognize the political realities that may make this difficult. Where would the responsibility of such a fund stop, given the importance of the wider determinants of health? At this stage, these issues must be raised but cannot be resolved.

Beyond the practical challenges of implementation, we can expect many principled objections to such a fund, but we believe they can be rebutted:

• A global social health protection fund would make existing institutions such as WHO or the World Bank redundant? The main roles of the WHO and the World Bank are to provide technical assistance and set priorities. Separating financing and analysis could resolve potential conflicts of interests. In the future, their financial roles are likely to remain limited.

• Aid would become invisible or anonymous within a global pool, and impede the ability to demonstrate tangible results? Standardizing reporting and delivery mechanisms will enable more effective analysis and learning from aid success and failure. Similar to pooling mechanisms within the World Bank and Global Fund, donors can claim credit for their contributions. Private donations would also continue to exist alongside the global social health protection fund.

• Global redistribution will distort markets? A global social health protection fund would correct market failures, and provide goods and services that have positive externalities.

• The Global South will forever rely on Global North? Mutual interdependency already exists in markets, climate change, and global processes. A global social health protection fund can redress adverse dependency, as resources flow from the South to the North. Over time, countries can graduate to positions where they no long require assistance, but rather provide it to those with greater needs. This has already occurred when borrowing countries from the International Development Association of the World Bank became ineligible for loans but now are donors.

A global social health protection fund challenges the fundamental paradigm underpinning global efforts to achieve the MDGs. Like any international development program, the MDGs are rooted in a particular paradigm--a series of prescriptive assumptions, often implicit, about how people in different parts of the world should assist each other (or not) in realizing their full potential as human beings and as societies. The paradigm that has been adopted since the adoption of the MDGs was the idea that a Big Push out of poverty traps could be achieved by a temporary influx of aid, in line with the model of welfare states within nations and free trade between them, leading to economic convergence. Unfortunately, recent evidence has shown that such an influx could not overcome the permanent forces that continue to push populations into poverty traps [27]. In response, just as states have created within-country mechanisms to redistribute capital as a means of insuring those who are at the losing end of the economic marketplace, so we can create a system of protection that can travel across borders to counterbalance the resources and labor costs that travel from poor to rich countries.

As with any international effort, the precise mechanisms and effects of a global social health protection fund cannot be known until one is formulated and implemented, but the failures of the existing approach suggest that a reliable, coordinated and permanent mechanism for the redistribution of wealth will be needed to address the shortcomings of the present international aid paradigm that have hampered efforts to achieve the MDGs. In addition, such a fund has the potential to rectify ongoing ethical dilemmas created by current models of trade. Much as the Global Fund redefined notions of sustainability for infectious disease programs, we believe that a sustained redistribution of wealth through a global social health protection fund could form one rational approach to overall improvement of development assistance for health.

## Competing interests

The authors declare that they have no competing interests.

## Authors' contributions

GO, DS, SB and MM have equally contributed to this paper. All authors have read and approved the final manuscript.
